# Effect of Injection Parameters on the MRI and Dielectric Properties of Condensation-Cured Silicone

**DOI:** 10.3390/polym15244670

**Published:** 2023-12-11

**Authors:** Conor Cristant, Kamal Kolasangiani, Siddharth Sadanand, Habiba Bougherara, Dafna Sussman

**Affiliations:** 1 Department of Electrical, Computer and Biomedical Engineering, Toronto Metropolitan University, Toronto, ON M5B 2K3, Canada; ccristant@torontomu.ca (C.C.); ssadanand@torontomu.ca (S.S.); 2 Institute for Biomedical Engineering, Science and Technology (iBEST), St. Michael’s Hospital & Toronto Metropolitan University, Toronto, ON M5B 1T8, Canada; kamal1.kolasangiani@torontomu.ca (K.K.); habiba.bougherara@torontomu.ca (H.B.); 3 Department of Mechanical and Industrial Engineering, Toronto Metropolitan University, Toronto, ON M5B 2K3, Canada; 4 Department of Obstetrics and Gynaecology, University of Toronto, Toronto, ON M5G 1E2, Canada

**Keywords:** cured silicone, custom-made injection, extrusion parameters, MRI properties, void content, dielectric properties

## Abstract

Phantoms with tissue-mimicking properties play a crucial role in the calibration of medical imaging modalities, including Magnetic Resonance Imaging (MRI). Among these phantoms, silicone-based ones are widely used due to their long-term stability in MR imaging. Most of these phantoms are manufactured using traditional pour-mold techniques which often result in the production of air bubbles that can damage the phantom. This research investigates the feasibility of utilizing extrusion techniques to fabricate silicone phantoms and explores the effects of extrusion parameters including plunger speed and nozzle diameter on void content, T1 and T2 relaxation times, and dielectric properties. A custom double-syringe silicone extrusion apparatus was developed to prepare the silicone samples. The void content, relaxometry, and dielectric properties of extruded samples were measured and compared with traditional poured samples. The results show that extrusion parameters can affect the void content of the silicone samples. The presence of voids in the samples resulted in lower T1 values, indicating an inverse relationship between void content and relaxometry. This study demonstrates the potential of extrusion techniques for manufacturing silicone phantoms with reduced air bubble formation and provides valuable insights into the relationship between extrusion parameters and phantom properties.

## 1. Introduction

In the medical field, phantoms serve as artificial representations of human body structures. They play a vital role in calibrating and assessing the performance of imaging systems prior to conducting scans on human bodies. Among them, Magnetic Resonance Imaging (MRI) phantoms are extensively utilized for various purposes, including the calibration and verification of MRI equipment, the advancement of new systems and pulse sequences, and training MRI operators. Silicone represents one of the predominant materials currently employed for fabricating phantoms in the field of research and experimentation. The utilization of silicone-based phantoms, with their remarkable attributes such as structural stability, stretchability, extended shelf life, and ease of manipulation, coupled with their capability to facilitate the dynamic imaging of physiological processes in motion, is gaining significant traction and contributing to their growing popularity [1,2]. The development of high-quality tissue-mimicking silicone phantoms holds high importance in the discipline of preclinical testing for emerging imaging and diagnostic modalities [3]. 

The current approach employed in phantom manufacturing involves the utilization of a custom plastic mold designed to match the desired 3D structure. The process entails pouring the silicone material into the mold and allowing it to undergo the curing and solidification process [4,5,6]. The issue with this technique is that it is both slow and suboptimal, as the pouring variability of the silicone leads to air bubbles and voids in the final product [7]. These bubbles can negatively impact the tactile, mechanical, and imaging properties of the phantom, and increase susceptibility to cracks, lowering the longevity of the phantom, especially for MRI applications [7,8]. Such voids can also cause reflection or artifacting in certain imaging modalities [8]. Due to silicone’s high viscosity, it is very difficult to extract the air bubbles created once solidification starts. 

One potential approach to mitigate the presence of air bubbles involves the utilization of a high-pressure vacuum chamber coupled with a periodic degassing technique. This method entails subjecting the phantom to a vacuum environment prior to complete cross-linking for a specific duration, thereby allowing the ascent of entrapped air to the surface [9]. Once the vacuum is turned off, it allows the silicone material to return to its normal/original state. However, since the entire phantom needs to be inserted into the vacuum chamber, this approach requires equally large vacuum tanks. Even when an appropriate chamber size is available, the process of vacuuming out air bubbles is still suboptimal because the silicone typically solidifies faster than air bubbles can be vacuumed out. The alternative method of constructing the phantom in smaller segments presents certain drawbacks as well. This approach necessitates the subsequent reassembly of the segmented parts using adhesive materials, which inherently lack the identical mechanical and electromagnetic properties of the phantom material. Consequently, this can lead to the appearance of visible seam lines in the resulting images of the final assembled phantom.

The extrusion techniques, as a promising alternative method, can be used for the manufacturing of the phantom. Variations in injection parameters such as plunger speed and nozzle diameter lead to changes in air bubbles and voids. From the literature, it was found that the presence of air influences relation times, with the T1 relaxation time decreasing as the volume of trapped air increases [10,11,12,13,14]. This research aims to investigate the feasibility of utilizing extrusion techniques in the manufacturing of silicone phantoms, to mitigate the occurrence of air bubbles by circumventing conventional pouring methodologies. Additionally, our study intends to explore the effect of extrusion velocity and the nozzle diameter of the syringes employed during the silicone extrusion process on the void content, MRI, and dielectric properties of the extruded silicone samples. To accomplish this objective, a tailor-made double-syringe silicone extrusion apparatus was developed, and the properties of the extruded silicone were characterized and compared with traditional poured samples. The research found a noticeable correlation between the longitudinal relaxation time (T1) and the void content observed within the silicone samples.

## 2. Materials and Methods

### 2.1. Material Specification and Preparation of Silicone Gel

The silicone material used in this study is condensation-cured silicone rubber (Mold Max XLS II, Smooth-On Inc., Easton, PA, USA). The silicone material was supplied in a liquid state, divided into two parts, referred to as Components A and B. These components were combined in a volumetric ratio of 10:1, respectively, followed by the manufacturer’s recommended stirring time of 3 min before the silicone was used in experimentation. The properties of silicone rubber are shown in Table 1.

### 2.2. Injection Setup and Process for Preparation of Samples

**Figure 1:**
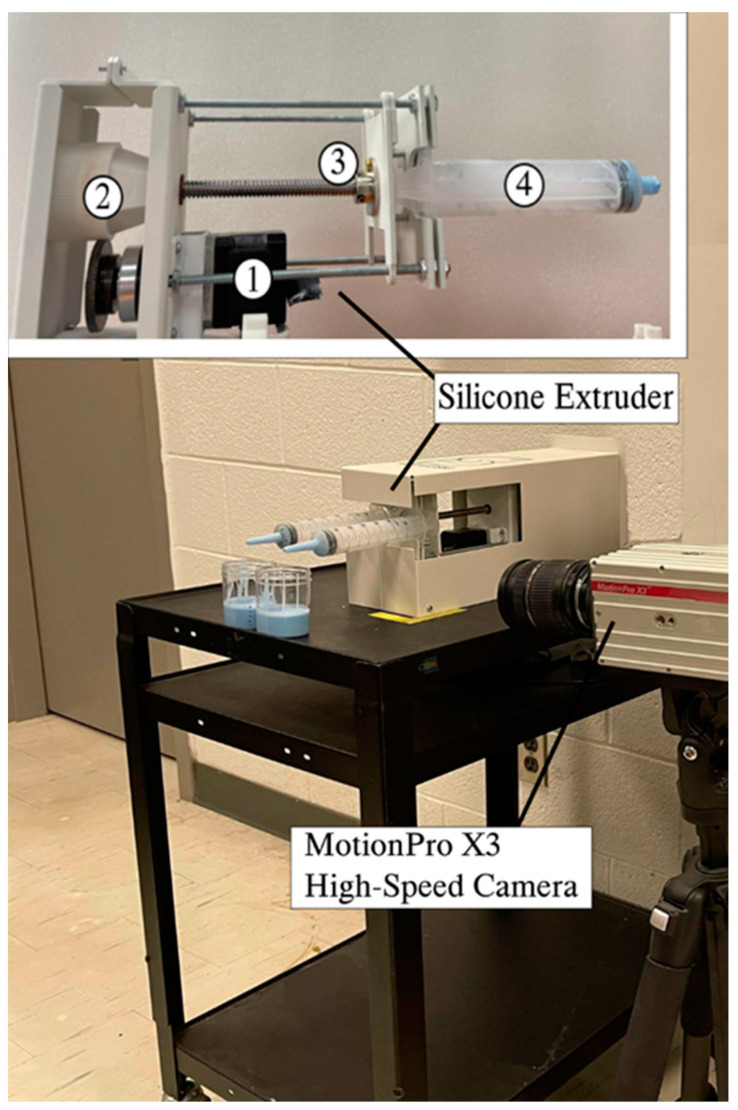
Experimental setup for extrusion process, including double-syringe silicone extruder, and MotionPro X3 high-speed camera.

The injected silicone samples were made using a dual-syringe silicone extrusion apparatus (see Figure 1). This system consists of four parts: (1) a stepper motor, (2) a gearbox with a gear ratio of 1:1 including a driver gear (SS0.5-84A, KHKGEARS, Mineola, NY, USA) and a driven gear, with a module of 0.5 and a 20o tooth pressure angle that was fabricated in PLA by a Fused Deposition Modeling (FDM) 3D printer, (3) a threaded screw, and (4) double syringes. In this extrusion system, the gears are driven by the programmed stepper motor, which provides a linear motion through the threaded screw, pushing the syringe plungers. 

A high-speed camera (Motion Pro X3, RedLake, San Diego, CA, USA) was positioned at the side of the extrusion unit directly in the line of sight of the syringes. The camera was connected to a laptop running the MotionStudio x64 software (Integrated Design Tools, Pasadena, CA, USA) in order to record at a slow frequency of 100 Hz. The purpose of the camera was to document the speed of each silicone extrusion and verify that all extrusions were performed at identical speeds, regardless of the extrusion nozzle diameter. The Tracker software version 5.1.5 (Open-Source Physics, Open Source) was employed to analyze the footage captured by the high-speed camera and compute the pixels moved per second, from which the speed was derived.

After the silicone was mixed according to the process described in Section 2.1, it was inserted into the extrusion cylindrical syringes. The selected extrusion speed was used to horizontally inject 50 mL of the sample into 64 jars (ULINE Wide Mouth Jars), with two jars being injected at a time. The specimens were cured at room temperature for 24 h and then post-cured at 65 °C in an oven for 4 h to remove any residual by-products generated during the condensation reaction. In total, 64 samples were made using injection technique.

### 2.3. Traditional Method for Preparation of Samples

The preliminary process to the traditional method follows the same preparation method outlined in 2.1. The traditional approach, however, involves the deposition of samples through a pouring process, rather than extrusion. The process involves separate 50 mL samples being injected into jar vessels, with 10 mL of silicone being injected at a time. After each 10 mL segment of the sample was poured, the sample was subjected to a cyclic vacuum treatment in a five-gallon vacuum chamber (Smooth-On Inc., Easton, PA, USA). This cyclic vacuuming process was performed in order to bring air bubbles to the surface of the sample, removing them from the sample to decrease the sample porosity. This step was repeated until each sample reached 50 mL. The specimens then underwent a curing process at room temperature for 24 h. An additional post-curing step was performed at 65 °C for 4 h to remove any residual by-products generated during the condensation reaction, including water and alcohol which may hinder the complete curing of the material. In total, four samples were made using the traditional technique.

### 2.4. Dielectric and MRI Measurements of Condensation-Cured Silicone

MRI testing was conducted on the specimens using the Siemens Magnetom Prisma 3T system. The system is equipped with a standard multinuclear configuration consisting of 2RF 8μT amplifiers, 80 mT/m gradients, and 200 T/m/s slew rate. To facilitate the imaging, the 18-channel circularly polarized body flex coils were employed. An Inversion Recovery sequence and a Multi Echo Spin Echo Sequence were used to measure the T1 and T2 relaxation values, respectively. The Inversion Recovery sequence utilized varied inversion times (TI) of 23, 150, 250, 500, 1000, 2000, and 4000 ms, while maintaining a fixed echo time (TE) of 8.1 ms and a repetition time (TR) of 9000 ms. The multi-echo sequence incorporated a range of echo times (TE) of 8.5, 17, 25.5, 34, 42.5, 51, 59.5, 68, 76.5, 85, 93.5, 102, 110.5, 119, 127.5, 136, 144.5, 153, 161.5, 170, 178.5, 187, 195.5, 204, 212.5, 221, 229.5, 238, 246.5, 255, 263.5, and 272 ms, alongside a fixed repetition time (TR) of 500 ms. The acquired imaging data were subjected to analysis through the utilization of the Segment software version 4.0 R11044c (Medviso, Lund, Sweden). This software facilitated the computation of T1 and T2 relaxation values for the samples, focusing specifically on user-defined regions of interest (ROIs). The ROIs were strategically chosen to isolate and characterize the relaxometry properties of the silicone, thereby eliminating any potential signal contamination originating from the sample containers. The equations used to calculate the T1 and T2 values are as follows:

S = M_0_ [1 − exp(−TR/T1)], (1)

S = M_0_ exp(−TE/T2), (2)

where S represents the signal intensity, M_0_ denotes the initial magnetization, TR signifies the repetition time, TE indicates the echo time, and T1 and T2 stand for the longitudinal and transverse relaxation times, respectively.

To evaluate the dielectric measurements, a dielectric assessment kit system (DAKS-12 & R60, SPEAG Schmidt and Partners Engineering AG, Zürich, Switzerland) was used to measure the conductivity (σ) and dielectric constant (ε′) of each sample. These measurements were taken at a frequency range from 63 to 315 MHz with a step size of 2 MHz. The DAKS probe allowed for the extraction of the dielectric constant (ε′) and dielectric loss (ε″) data. The conductivities of each sample were determined using Equation (3), where ⍵ represents the angular frequency (⍵ = γB), γ is the gyromagnetic ratio (γ = 42.57747 × 10^6^ rad⋅s^−1^⋅T^−1^), B denotes the magnetic field strength (B = 3 T), ɛo represents the permittivity of free space (ɛo = 8.85 × 10–12 F/m), and tan δ is the loss tangent (tan δ = ɛ″/ɛ′). All samples had exactly 50 mL of silicone extruded or poured into the same wide mouth jars, meaning the sample thickness was a standard 24 mm thick to ensure a minimization of stray capacitance affecting the dielectric results. The resulting data were recorded and organized into tabular and graphical forms.

Σ = ε_0_ × ω × ε′ × tan δ, (3)

### 2.5. Processing Parameters 

Two independent injection parameters, namely the speed of the plunger from the syringe through the extrusion mechanism and the diameter of the extrusion nozzle, were selected. The effect of these parameters on the dielectric and MRI properties (T1 and T2 relaxation times) as well as the void content were investigated. A range of extrusion nozzle diameters was tested: d1 = 3.8, d2 = 5.1, d3 = 6.3, and d4 = 7.8 mm, each with extrusion speeds of 0.023, 0.048, 0.071, and 0.097 mL/s, leading to sixteen distinct experimental conditions. Each processing condition was repeated four times, leading to a total of 64 samples.

### 2.6. Image Analysis

**Figure 2:**
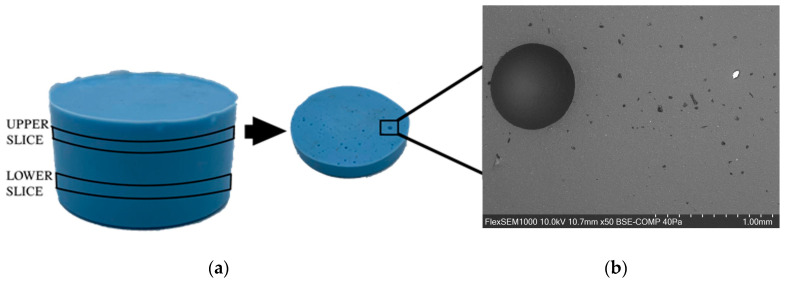
(**a**) Location of the upper and lower slices on the silicone sample, (**b**) upper slice of silicone sample and the SEM view of a section of the upper slice, showcasing a spherical void.

To perform void volume fraction analysis, the samples were sectioned horizontally to expose the internal surface areas. For the two nozzle diameters of interest, two samples per extrusion speed were prepared for image analysis, with an additional two samples made using the conventional fabrication method. This resulted in a total of 18 samples used in the image analysis. Each sample was sliced twice, horizontally, into an upper (U) and lower (L) slice, at 8 mm from the top and bottom of the sample. The two surface faces used for the image analysis were the lower face of the top horizontal section, and the upper face of the lower horizontal section, as shown in Figure 2a. 

The porosity of each surface face was analyzed using a scanning electron microscope (Hitachi Flex SEM 1000II, Chiyoda City, Tokyo, Japan) to detect any pores present at different planes of imaging. Each face was imaged 10 times to cover the entire surface area with each image taken at 50× magnification. The ImageJ software version 1.53t (National Institutes of Health, Open Source) was used to process each image by automatically normalizing the brightness/contrast to reveal all pores in the image area, and the porosity percentage was automatically measured by the software.

## 3. Results and Analysis

### 3.1. Effects of Processing Parameters on Void Volume Fraction

The area enclosed by the black rectangle on the upper slice (Figure 2a) was magnified using SEM and shown on the right (Figure 2b) for further inspection in order to estimate the void content. The SEM image reveals the presence of significant large and small voids at various locations on the slice surfaces. 

**Figure 3:**
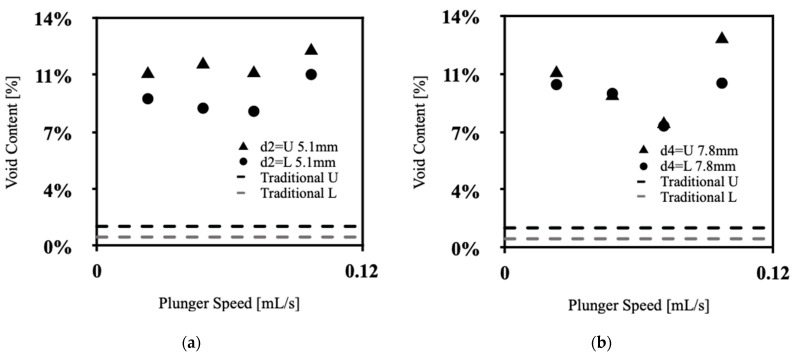
Void content of upper and lower slices of silicone samples versus plunger speed (**a**) d = 5.1 mm, (**b**) d = 7.8 mm.

Figure 3 presents the percentage of voids on the upper and lower slices of the injected silicone samples as a function of the syringe plunger speed for the nozzle diameters of 5.1 and 7.8 mm and traditional samples. The void content on the upper and lower slice surfaces of the injected specimens shows a decreasing trend as a function of plunger speed until reaching 0.048 and 0.071 mL/sec followed by a sudden increase at 0.071 and 0.097 mL/sec for the diameters of 5.1 and 7.8 mm, respectively. The higher void content observed at initial speeds can be attributed to the discontinuity that occurs in the downward flow stream from the nozzle. In all cases, the Reynolds number at the minimum point of void content is small enough to assume that the flow motion from the nozzle is laminar. The sudden increase in void content after the minimum point is caused by the acceleration of the silicone falling onto the free surface. This acceleration is proportional to the entrapment of air bubbles, which occurs when the surface tension equals the dynamic pressure in the flow [16]. Air density is approximated to 0 because it is nearly 1000 times less than water. The higher void content on the upper slice in most plunger speeds can be attributed to the movement of the bubbles with zero density to the upper part during the curing process. The lowest void content was achieved in the traditional samples due to employing the periodic degassing method during sample preparation. Furthermore, an examination of Figure 3a reveals that the void content values exhibit less variability when the plunger speed is altered for the smaller extrusion diameter of 5.1 mm, as compared to the larger extrusion diameter of 7.8 mm shown in Figure 3b. This observation suggests a potential pattern indicating that the void content output in extruded samples is less influenced by changes in plunger speed for smaller extrusion diameters.

### 3.2. Effects of Processing Parameters on MRI Relaxometry Properties

**Figure 4:**
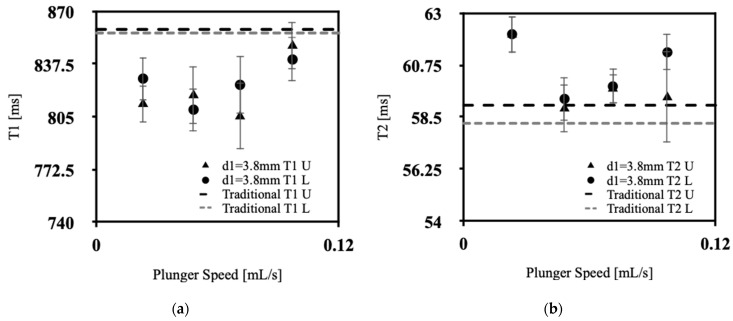
Relaxometry properties of silicone samples versus plunger speed for d1 = 3.8 mm, (**a**) T1, (**b**) T2.

**Figure 5:**
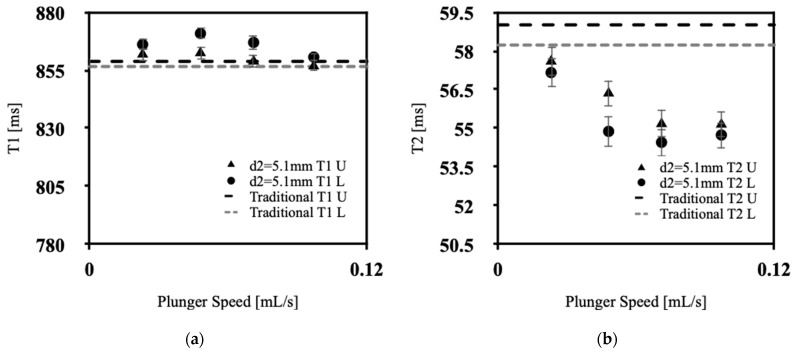
Relaxometry properties of silicone samples versus plunger speed for d2 = 5.1 mm, (**a**) T1, (**b**) T2.

**Figure 6:**
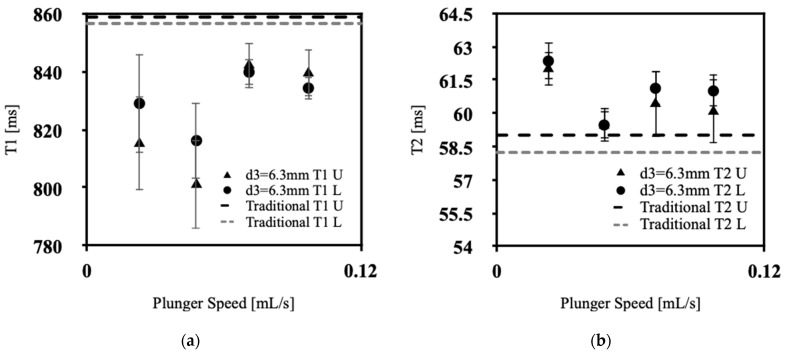
Relaxometry properties of silicone samples versus plunger speed for d3 = 6.3 mm, (**a**) T1, (**b**) T2.

**Figure 7:**
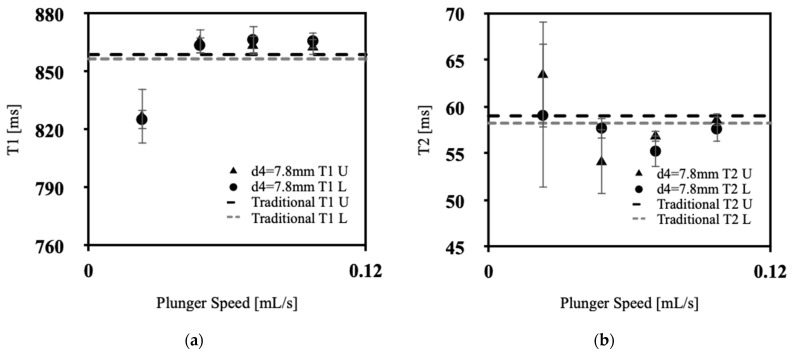
Relaxometry properties of silicone samples versus plunger speed for d4 = 7.8 mm, (**a**) T1, (**b**) T2.

Figure 4a, Figure 5a, Figure 6a and Figure 7a showcases the T1 values obtained across various extrusion speeds, with each graph representing data collected at distinct extrusion diameters, while Figure 4b, Figure 5b, Figure 6b and Figure 7b exhibit the corresponding T2 data for the respective extrusion diameters. The graphs distinguish the relaxometry data between the upper and lower slices of the silicone samples. In the relaxometry investigation of the condensation-cured silicone rubber samples, the T1 values ranged from 790 to 872 ms, while the T2 values ranged from 53.5 to 64.00 ms. 

There was no discernible correlation between the relaxometry properties (T1 and T2) and the extrusion speed of silicone. Similarly, no relationship was observed between the relaxometry properties and the diameter of extrusion at the nozzle tip of the silicone extrusion device. However, the data show lower T1 values at initial extrusion speeds, with a sharp increase for speeds between 0.048 and 0.071 mL/s, with a gradual decrease back to low T1 values at 0.097 mL/s, the exception being at an extrusion diameter of 3.8 mm. This pattern corresponds with the lowest percentage of sample porosity, in Figure 3, which was found to occur for extrusion speeds of 0.048–0.071 mL/s. As depicted in Figure 4a, Figure 5a, Figure 6a and Figure 7a, the upper slices consistently exhibit lower T1 values compared with the lower slices. This observation signifies an inverse relationship between the longitudinal relaxation of condensation-cured silicone and the presence of voids. Furthermore, the extruded silicone exhibits lower average T1 values in contrast to the silicone samples formed using the traditional method. This dissimilarity can be attributed to the inclusion of cyclic vacuuming in the traditional method, which facilitates the removal of a significant portion of the voids present in the samples. This phenomenon can be attributed to the disparity in T1 values between hydrogen protons in air and hydrogen protons within the silicone material. Protons in air demonstrate lower T1 values relative to hydrogen protons in silicone. Consequently, samples characterized by a higher void content will inherently yield lower overall T1 values. This relationship is further supported by the work of Alamidi et al. [10], who conducted a study on the measurement of T1 values in individuals diagnosed with Chronic Obstructive Pulmonary Disease (COPD). Their findings revealed that patients with COPD exhibited notably lower T1 values in comparison to those with healthy lung function. The decreased T1 values observed in COPD subjects can be attributed to the increased air volume present in their lungs, resulting from the disease-induced restrictions on inhalation and exhalation [11]. Additional studies conducted by Naish et al. [12] and McGrath et al. [13] explored the impact of inhaling regular air versus hyperoxic inhalation on the longitudinal relaxation (T1) of the lungs and muscles, respectively. Both studies consistently noted that the tissues under examination exhibited lower T1 values when exposed to a hyperoxic environment [12,13]. This phenomenon can be explained by the inherent contrast between the T1 values between pure oxygen and standard air. Oxygen-rich pockets within the tissue naturally have lower T1 values compared to those associated with standard air [14]. Therefore, it can be inferred that when measuring T1 values in samples characterized by high porosity and a significant amount of trapped air, the overall T1 value of the sample diminishes as the void content increases.

From Figure 4b, Figure 5b, Figure 6b and Figure 7b, the T2 values of the extruded silicone and the silicone produced via the traditional method exhibit closer proximity. This phenomenon can be explained by the relatively smaller difference between the T2 values of air and silicone compared to the difference between their respective T1 values. This observation indicates that the presence of air exerts a lesser impact on altering T2 values.

**Figure 8:**
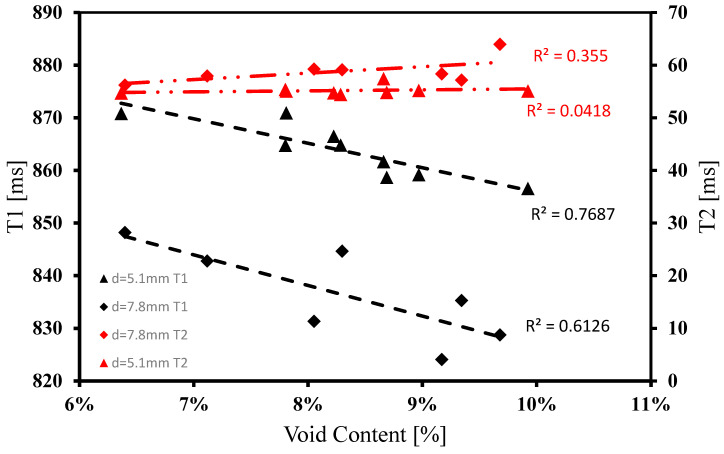
R-squared statistical analysis of T1 and T2 versus void content for nozzle diameters d2 = 5.1 and d4 = 7.8 mm.

The statistical significance of the observed data patterns was assessed through two significance tests, namely, R-squared (R²) testing and Pearson’s test of correlation. To investigate the correlation between the T1 and T2 relaxation properties of each silicone sample and its corresponding void content, scatter plots were constructed. In order to assess the goodness of fit in each data set, the coefficient of determination (R-squared, R²) was computed and plotted alongside their corresponding linear trendlines. These plots were amalgamated in Figure 8 to facilitate a direct visual comparison of the slopes, aiding in the subsequent R-squared statistical analysis.

The analysis of R-squared values reveals that the T1 data demonstrate a relatively low variance compared to a linear trend, with values of 0.769 and 0.612 for the 5.1 mm and 7.8 mm diameter data, respectively. Conversely, the T2 data exhibit a higher variance when compared to a linear trend, with values of 0.0418 and 0.355 for the 5.1 mm and 7.8 mm diameter data, respectively. This indicates that the T2 data deviate more substantially from its linear trend line in comparison to the T1 data, suggesting a weaker likelihood of a linear relationship. It is important to note that R2 values exceeding 0.7 generally indicate a good linear fit of the data, a criterion that applies to both sets of T1 data. The decreasing trend indicates an inverse relationship. 

The sample groups within each data set, plotted in Figure 8, were subjected to Pearson’s test of correlation, aiming to determine the existence of a statistically significant correlation between the relaxometry and void content data derived from each sample slice. The correlation analysis was executed utilizing the Data Analysis built-in correlation test feature in Microsoft Excel (Microsoft, Redmond, Washington), which employs the Pearson’s test of correlation equation as described by Equation (4) [17], where r represents Pearson’s correlation coefficient, x and y indicate relaxometry properties (T1 and T2) and void content, respectively. cov represents the covariance between the dataset’s corresponding relaxometry and void content values, and σx and σy represents the standard deviation of the X or Y dataset respectively.

r_XY_ = (cov(X,Y))/(σ_X_·σ_Y_), (4)

The Pearson’s correlation coefficient values echo the statistical trends observed in the R-squared data. This coefficient serves as a measure to assess the strength and direction of the linear relationship between two continuous datasets. The T1 data have a strong negative correlation, indicative of a decreasing pattern, with correlation coefficients of −0.877 and −0.783 for the 5.1 mm and 7.8 mm diameter data, respectively. On the other hand, T2 data exhibit a low correlation strength, but in the positive direction, with coefficients of 0.204 and 0.596 for the 5.1 mm and 7.8 mm diameter data, respectively. Both statistical tests indicate a pronounced linear correlation in the decreasing pattern for the T1 data, while there is uncertainty in the correlation of the T2 data.

The discrepancy in the impact of void content on T1 relaxation compared to T2 relaxation can be attributed to a fundamental argument of means. Considering that the T2 of air at 3T is approximately 0.5 ms, which can be assumed to be very close to the T1 value of air at the same magnetic field strength, it is evident that air possesses relatively short relaxation times. In contrast, the average T1 value of silicone in traditionally poured samples is approximately 860 ms, while the T2 value is around 59 ms. Traditional pouring methods are employed because they yield samples with the lowest void content, thereby containing the highest silicone content per sample. A detailed analysis reveals that the change in mean T2 between 0% void fraction and 10% void fraction is approximately 6 ms, whereas the change in mean T1 between 0% void fraction and 10% void fraction is about 86 ms. This represents an almost 80 ms increase in variance. Consequently, any alteration in void content significantly impacts the resulting T1 value but has a relatively lesser effect on the T2 value of the same sample. The relatively high variance in T1 compared to T2 can be attributed to the substantial difference in relaxation times between air and silicone, resulting in a more pronounced sensitivity of T1 to variations in void content.

### 3.3. Effects of Processing Parameters on Dielectric Properties

**Figure 9:**
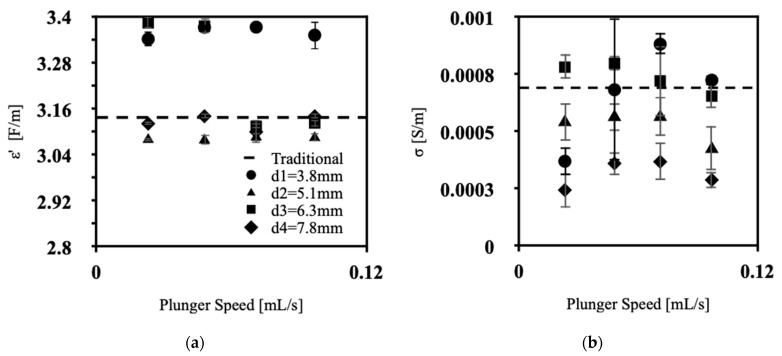
Dielectric properties of silicone samples versus plunger speed (**a**) dielectric constant, (**b**) conductivity.

The dielectric constant and conductivity measurements were conducted at 127 MHz, the resonant frequency of hydrogen protons in a 3T MRI system [18]. While the Siemens Prisma is marketed as a 3T clinical system, the measured static field is B_0_ = 2.89 T, with a resultantly lower resonant frequency of 123 MHz. Nonetheless, the dielectric property measurements in this work are expected to be similar to those applicable to sub-3T systems marketed as 3T. Figure 9a showcases the dielectric constant values obtained across various extrusion speeds for all nozzle diameters. Similarly, Figure 9b shows the corresponding conductivity data for the respective extrusion diameters. As shown in the graphs, the dielectric constant ranged from 3.06 to 3.40 F/m, while the conductivity ranged from 0.000193 to 0.001003 S/m.

As seen in Figure 9b, lower conductivity values were obtained at lower initial extrusion speeds. Subsequently, there is a notable increase in conductivity within the speed range of 0.048–0.071 mL/s, followed by a gradual decline, ultimately returning to low conductivity values at 0.097 mL/s. Figure 9b shows a trend that closely mirrors the inverse of the void content depicted in Figure 3a–b, highlighting the influence of air on sample conductivity due to its extremely poor conductivity [19]. Furthermore, the traditional silicone displays higher values of conductivity compared to the mean of the extruded silicone samples. This too may arise from reduced air voids in the traditional samples achieved by cyclic vacuuming. These findings highlight the need for continued investigation of methods to reduce silicone phantom material air void content.

Concerning the dielectric constant (Figure 9a), samples with an extrusion diameter of 3.8 mm and 6.3 mm, lie above the traditional value, while samples with extrusion nozzle diameters of 5.1mm and 7.8mm are similar to the dielectric constant of the poured silicone. Stray capacitance may have been a significant confound in these measurements, effacing any apparent significant trend [20]. This confound, however, would manifest consistently across all samples under identical experimental conditions, with apparent trends in measurements and their correlates are real phenomena. 

## 4. Conclusions and Recommendations

In this paper, the effect of injection parameters on the void content, MRI, and dielectric properties of condensation-cured silicone was examined and compared with traditional poured samples. A custom double-syringe silicone extrusion apparatus was developed to prepare extruded silicone samples. The results showed that the injection parameters (i.e., extrusion velocity and nozzle diameter) affected the void content of the samples, which in turn can affect certain relaxometry and dielectric properties. MRI relaxometry analysis unveiled that the extrusion speed, rather than the extrusion diameter, exerted a discernible influence on T1 relaxometry. The data demonstrate lower T1 values observed at lower extrusion velocities, contrasted with a notable escalation within the speed range of 0.048–0.071 mL/s. This phenomenon was attributed to a reduction in void content within that specific range. Conversely, no discernible pattern was observed in the T2 relaxation data. Furthermore, the dielectric analysis of silicone samples revealed that extrusion speed exhibited a discernible influence on conductivity. Notably, within the extrusion speed range of 0.048–0.071 mL/s, conductivity attained its peak value across all extrusion diameters. This can be attributed to the minimized void content within these speed ranges, as air possesses significantly poor conductivity. However, the systematic alteration of extrusion parameters during the experimental testing did not produce any conspicuous pattern in the dielectric constant.

In comparison to conventional outcomes, the extrusion of silicone using the experimental setup does not yield the same low void content as the traditional pouring method combined with cyclic vacuuming. The findings of this study highlight the importance of carefully controlling the injection parameters during the fabrication of silicone phantoms to minimize the occurrence of air bubbles and optimize the imaging properties. Corrective measures should be taken to address the issues of the experimental setup, specifically mitigating the impinging effect of silicone on the base of the sample jar during extrusion. Furthermore, it is recommended to conduct further scanning electron microscopy (SEM) analyses on silicone samples extruded at additional speeds within the range of 0.048–0.071 mL/s to precisely determine the extrusion speed that yields the lowest possible void content.

The utilization of the extrusion technique presents a promising alternative to the conventional pouring method, offering an efficient means of generating high-quality silicone phantoms. However, further investigations are warranted to optimize the positioning of the extruder and the spatial relationship between the extruder and the mold. Furthermore, it is recommended to incorporate the tested extruder into a custom-designed Fused Deposition Modeling (FDM) 3D printer or a comparable apparatus capable of facilitating extruder movement. This setup would enable the comprehensive evaluation of silicone extrusion within an authentic phantom context, thereby enhancing the practical applicability of the findings.

## Figures and Tables

**Table 1:** Technical properties of condensation-cured silicone, as provided by Smooth-On Inc. [15].

	Mixed Viscosity [cp]	Specific Gravity [g/cc]	Specific Vol. [cu.in. /lb.]	Pot Life [min]	Cure Time [h]	Shore Hardness	Tensile Strength [psi]	100% Modulus [psi]	Elongation at Break [%]	Die B Tear Strength [pli]	Shrinkage [in./in.]
**Mold Max XLS II**	30,000	1.22	22.7	40	24	30	550	95	375	110	0.001

## Data Availability

The data presented in this study are available on request from the corresponding author.
